# Face the Pain: Radiobiological and Clinical Considerations of Re-radiosurgery to the Trigeminal Nerve Following Irradiation of an Abutting Petroclival Meningioma

**DOI:** 10.7759/cureus.84281

**Published:** 2025-05-17

**Authors:** Mariana Dejuk, James McInerney, Joseph A Miccio, Nicholas J Potter, Jeffrey M Ryckman, Jonathan Knisely, Sean Mahase

**Affiliations:** 1 Medicine, Penn State College of Medicine, Hershey, USA; 2 Neurosurgery, Penn State Cancer Institute, Hershey, USA; 3 Radiation Oncology, Penn State Cancer Institute, Hershey, USA; 4 Radiation Oncology, Penn State Health Milton S. Hershey Medical Center, Hershey, USA; 5 Radiation Oncology, West Virginia University, Camden Clark Medical Center, Parkersburg, USA; 6 Radiation Oncology, NewYork Presbyterian/Weill Cornell Medical Center, New York, USA

**Keywords:** gamma knife radiosurgery, petroclival meningioma, reirradiation, stereotactic radiosurgery (srs), trigeminal nerve compression, trigeminal neuralgia

## Abstract

Trigeminal neuralgia is a common symptom of benign tumors compressing the trigeminal nerve, leading to debilitating pain and a devastating impact on quality of life. Stereotactic radiosurgery is a validated option for intracranial meningiomas with excellent tumor control rates that correlate with symptomatic improvement. Select cases with refractory or recurrent trigeminal neuralgia can benefit from a second treatment targeting the trigeminal nerve. We present a case of refractory trigeminal neuralgia secondary to compression of a previously irradiated petroclival meningioma successfully treated with a second radiosurgery course targeting cranial nerve V (CN V). Multidisciplinary considerations, patient-centered factors, radiobiological considerations, and technical challenges faced in the intracranial reirradiation setting when cumulative dose constraints are previously met or exceeded are discussed.

## Introduction

Cranial neuropathies are frequently associated with petroclival meningiomas, with cranial nerve V (CN V)-related symptoms (neuralgia, paresthesia, numbness), comprising approximately 40% of symptomatic cases [1]. The trigeminal root entry zone is particularly susceptible to compression and is a transition point from oligodendroglial to Schwann cell myelination. Compressed nerves undergo demyelination and repair, promoting hyperexcitable states and ephaptic transmission [2], manifest as pain [3]. Trigeminal neuralgia (TN), entailing intense, debilitating, episodic electric shock-like pain along the CN V distribution, significantly impacts quality of life and is a known cause of suicide. Pharmacological therapies, including carbamazepine and gabapentin, are first-line options providing variable effectiveness and duration and whose use is limited by tolerability [1,4]. Surgical considerations are challenging owing to anatomical considerations, including association with the brainstem, cranial nerves, and vasculature, as well as patient-specific factors. Stereotactic radiosurgery, a validated option for intracranial meningiomas with excellent tumor control rates, is also limited by the radiation tolerances of abutting organs at risk. Prior publications describing subsequent radiosurgery to the trigeminal nerve following irradiation of a petroclival tumor focus on clinical outcomes [1,5], rather than radiobiological considerations in the context of significant heterogeneity and dearth of data regarding reirradiation practices. Herein, we present a case of refractory TN secondary to compression of a previously irradiated petroclival meningioma successfully treated with radiosurgery targeting CN V and discuss the feasibility and safety of this approach.

## Case presentation

**Figure 1:**
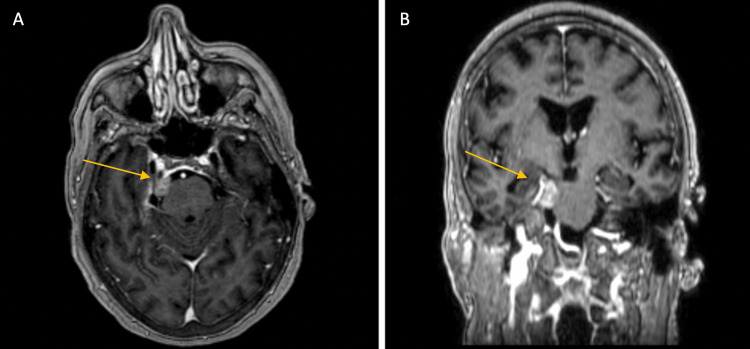
Contrast-enhanced MRI showing the right prepontine cistern lesion (A) axial and (B) coronal T1-weighted contrast-enhanced magnetic resonance imaging demonstrating an enhancing lesion in the right prepontine cistern along the tectorial incisura, superior to Meckel's cave, approximately 1.3 cm. This lesion abuts the cisternal segment of the right trigeminal nerve, which is deviated inferiorly.

An 81-year-old, right-handed woman presented with trigeminal neuralgia encompassing the right V2-V3 distribution. Her pain was successfully managed with carbamazepine. Following dental work, the pain resolved, allowing discontinuation of medication. Her symptoms returned four years later in the same distribution, exacerbated by yawning or chewing. She restarted carbamazepine 200 mg twice daily without relief. Subsequent brain MRI revealed a 1.6 x 1.0 x 1.5 cm dural-based mass, consistent with a meningioma, arising from the right tentorium, impinging on the right side of the pons and the superior aspect of the right fifth cranial nerve (Figures 1A-1B).

**Figure 2:**
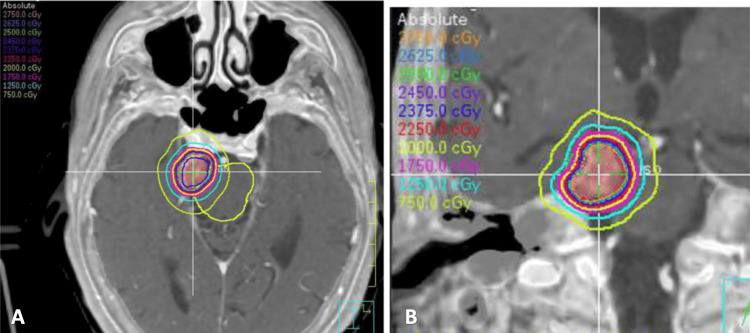
Views of linac-based fractionated radiosurgery plan with isodose lines (A) axial and (B) coronal images of patient’s linac-based fractionated radiosurgery plan. Isodose lines: light green, 7.5 Gy; teal, 12.5 Gy; magenta, 17.5 Gy; yellow, 20 Gy; red, 22.5 Gy; dark blue, 23.75 Gy; green, 25 Gy; light blue, 26.25 Gy; orange, 27.5 Gy

Her symptoms were attributed to the meningioma impinging on her right trigeminal nerve, and linac-based stereotactic radiosurgery (SRS) was recommended. Her meningioma was treated at an outside institution to 25 Gy in five fractions. Her brainstem received 31.0 Gy to 0.03 cc and 23.0 Gy to 0.5 cc (Figure 2). The trigeminal nerve received 35.6 Gy to 0.03 cc and 34.5 Gy to 0.5 cc.

While her meningioma remained stable in size on surveillance imaging, her trigeminal symptoms only transiently improved. Her facial pain persisted in the V2 and V3 distributions, exacerbated by chewing, talking, and emotional stress. One year after radiotherapy, she experienced an unrelated ischemic left middle cerebral artery stroke, resulting in expressive and receptive aphasia.

**Figure 3:**
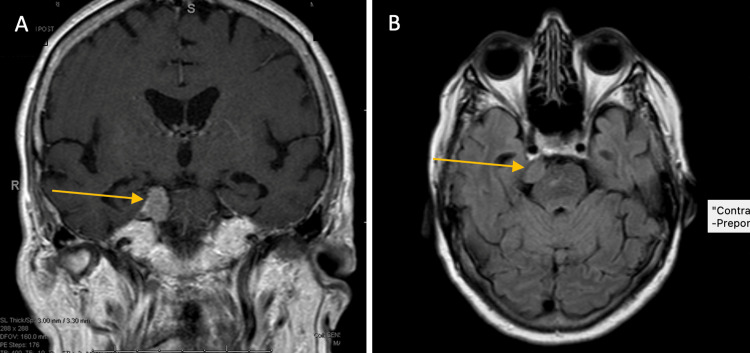
Surveillance brain MRI demonstrating stable cerebellopontine angle meningioma without edema (A) Coronal T1-weighted contrast-enhanced MRI demonstrated treated right cerebellopontine angle meningioma (yellow arrow), unchanged from pre-treatment. (B) Axial fluid-attenuated inversion recovery (FLAIR) MRI demonstrating no hyperintense signal in association with the treated meningioma (yellow arrow).

Her pain became refractory to medical management, prompting consideration of further intervention two years after completing radiotherapy. Her primary radiation oncologist referred her for consideration of gamma knife radiosurgery. She was subsequently seen by neurosurgery, radiation oncology, and discussed at the multidisciplinary neuro-oncology tumor board. Her MRI at this time demonstrated no change in the meningioma, and no increased T2 or fluid-attenuated inversion recovery (FLAIR) signal, suggestive of edema or inflammation (Figure 3). Due to these MRI findings, and her co-morbidities (diabetes, hypertension, and osteoporosis), a steroid challenge was not recommended. Given the complexities of this case, including her debilitating pain, performance status being prohibitive to surgical intervention, prior radiotherapy, and risk of brainstem injury, Gamma Knife radiosurgery to her right trigeminal nerve was recommended.

**Figure 4:**
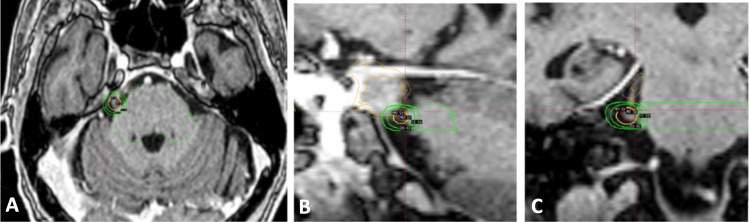
Views of gamma knife radiosurgery plan targeting the right trigeminal nerve Representative (A) axial, (B) sagittal, and (C) coronal T1-weighted contrast-enhanced MR image of single fraction gamma knife radiosurgery plan targeting the patient’s right trigeminal nerve. Yellow = 50% (40 Gy) isodose line, green = 20 Gy and 12 Gy isodose line, light green = brainstem, red = right trigeminal nerve, orange = meningioma + 1 mm margin.

She received 80.0 Gy prescribed to the maximum dose point using a 4 mm collimator to the right trigeminal nerve in one fraction (Figure 4). Her brainstem received 17.3 Gy to 0.03 cc and 4.1 Gy to 0.5 cc. The trigeminal nerve received 52.9 Gy to 0.03 cc, and the uninvolved nerve volume measured 0.037 cc. Tables 1-2 entail absolute doses to the brainstem right trigeminal nerve for each treatment course, respectively.

**Table 1:** Absolute doses to the brainstem and constraints to brainstem during RT1 and RT2 RT1 = first radiation course, RT2 = second radiation course, T = Timmerman tables 2021, UK = UK 2022 Consensus [6,7]

Variables	Fractions	Brainstem Dose Delivered		Brainstem Constraint	
		0.03 cc	0.5 cc	0.03 cc	0.5 cc
RT1	5	31.0 Gy	23.0 Gy	31 Gy ^TG-101, T, UK^	23 Gy ^TG-101, T, UK^
RT2	1	17.3 Gy	4.1 Gy	15 Gy ^TG-101, T, UK^	10 Gy ^TG-101, T, UK^

**Table 2:** Absolute doses to trigeminal nerve and constraints to brainstem during RT1 and RT2 RT1 = first radiation course, RT2 = second radiation course, N/A = not applicable, TGN = trigeminal, T = Timmerman tables 2021, *named nerve

Fractions	TGN Dose Delivered		TGN Constraint	
	0.03 cc	0.5 cc	0.03 cc	0.5 cc
5	35.6 Gy	34.5 Gy	38 Gy ^T^	N/A
1	52.9 Gy	N/A	N/A (20 Gy^T^)	N/A

The prior hypofractionated RT DICOM file was unavailable. However, as shown in Figure 2, the sample cross-sectional images illustrate that the trigeminal nerve courses adjacent to the tumor. Therefore, equivalent dose in 2 Gy/fraction (EQD2) calculations for each course and cumulative dose calculations (Table 3) assumed the most conservative scenario, where the highest dose was consistently delivered to the same region in each plan for both the trigeminal nerve and brainstem.

**Table 3:** EQD2 conversions for both the brainstem and trigeminal nerve RT1 = first radiation course, RT2 = second radiation course, N/A = not applicable, EQD2 = equivalent dose in 2 Gy/fraction.

Variables	Fractions	Brainstem		Trigeminal Nerve	
		0.03 cc	0.5 cc	0.03 cc	0.5 cc
RT1	5	57.04 Gy EQD2	31.92 Gy EQD2	72.05 Gy EQD2	68.31 Gy EQD2
RT2	1	70.24 Gy EQD2	5.82 Gy EQD2	591.42 Gy EQD2	N/A
RT1 + RT2		127.28 Gy EQD2	37.74 Gy EQD2	663.47 Gy EQD2	N/A

At her three-month follow-up, she endorsed symptomatic relief and was eating with less difficulty. She also noted improved response to carbamazepine. She remained asymptomatic one year after treatment.

## Discussion

This case underscores challenges in the reirradiation setting when cumulative dose constraints are met or exceeded with the first radiotherapy course. Prior publications on targeting the CN V for idiopathic trigeminal neuralgia suggest that isocenter proximity to the brainstem is associated with improved outcomes [8], supporting notions that the central oligodendrocyte myelin is more radiosensitive than more distal Schwann cells. The brainstem may receive varying amounts of radiation depending on patient anatomy and placement of the radiation dose. Accepted practice for radiosurgical plans for trigeminal neuralgia entails the 50% isodose line off of the brainstem, and the 30% isodose line tangential to, or covering <0.05 cc, of brainstem [9,10]. A review of over 100 trigeminal neuralgia cases treated to a median dose of 75 Gy reported a median brainstem dose of 15 Gy (range: 7.0-17.5 Gy). While the authors found no association between brainstem dose and side effects, they emphasized their current practice of ensuring the brainstem edge receives no higher than the 20% isodose line to minimize the theoretical radiation necrosis risk [11]. This suggests that historically defined brainstem constraints may be abundantly cautious, not reflecting contemporary advances in imaging, target delineation, and treatment delivery with modern radiosurgical platforms.

These considerations are emphasized in reirradiation settings. A retrospective analysis of 168 patients with petroclival meningiomas treated with radiosurgery demonstrated symptomatic improvement, stable neurological symptoms, and worsened or new neurological symptoms in 26%, 58%, and 15%, respectively. Patients with grade 1 meningiomas had 95% and 89% five- and 10-year progression-free survival rates, which correlated with symptom improvement or stability [1], supporting the initial approach targeting the meningioma. Huang et al. reported a 57% improvement in trigeminal neuralgia following radiosurgery for an abutting benign tumor, which increased 76% after symptomatically recurrent cases received a second treatment to CN V [5]. In our case, each course independently met or exceeded its respective absolute brainstem dose constraints before accounting for cumulative dose summation or conversion to EQD2. The recommended five-fraction radiotherapy brainstem dose constraints, 31 Gy to 0.03 cc and 23 Gy to 0.5 cc (Table 1), were achieved in the first radiotherapy course, but left no reserve for further treatment, assuming no normal tissue recovery. Even assuming 50% normal tissue recovery during the two-year interval between treatments, the brainstem single fraction dose constraint of 15 Gy to D0.03 cc was exceeded during RT2, where D0.03 cc was 17.3 Gy. Most publications describing repeated single-fraction radiosurgery for trigeminal neuralgia focus on clinical outcomes and cranial nerve toxicities, with the brainstem dose conscientiously minimized to avoid theoretical toxicity [12,13]. While concern is justifiably placed on providing symptomatic relief while minimizing cranial neuropathies, adhering to historical brainstem constraints may impact therapeutic efficacy. Furthermore, as in our case, there is little data guiding scheduling plans with differing dose-fractionation schemes.

The dearth of data on clinical reirradiation cases, where cumulative metrics may be exceeded within a single plan, is inherently challenging regardless of the anatomical site, instilling justifiable discomfort among physicians. Reirradiation practices are controversial due to a lack of available data and the lack of reliable methods to ensure reproducibility in planning, as there is no right way to plan each case. These cases continue to balance science with art, as physicians reasonably follow different practices based on first principles, personal risk tolerance and experience, hardware/software factors, and staff experience. This is underscored by reirradiation guidelines published by radiation societies deriving recommendations through panel discussions [14,15]. The creation of international registries to collect and analyze reirradiation metrics from multiple sources may provide sufficient power to provide modern normal tissue tolerance data.

In the context of the aforementioned considerations, it is ultimately the physician’s imperative to have a patient-centered discussion focusing on the uncertainty of the therapeutic ratio - balancing the risk of brainstem toxicity with therapeutic benefit. The patient experienced debilitating medication-refractory pain severely impacting her quality of life and associated with suicidal ideations. Furthermore, her comorbidities limited interventional options, leaving radiotherapy as the sole treatment option.

## Conclusions

This case underscores the multidisciplinary approach and radiobiological considerations entailing radiosurgery to CN V for trigeminal neuralgia following prior radiotherapy to a petroclival meningioma impinging CN V. Through careful consideration of patient history, imaging findings, and treatment options, personalized care was provided to optimize symptom relief and quality of life. Given the dearth of data and consensus on reirradiation considerations, these complicated scenarios should be approached on a case-by-case basis, carefully weighing clinical benefit against toxicity while ensuring the plan aligns with patient goals.
